# Implication of Electrophysiological Biomarkers in Psychosis: Focusing on Diagnosis and Treatment Response

**DOI:** 10.3390/jpm12010031

**Published:** 2022-01-02

**Authors:** Ho Sung Lee, Ji Sun Kim

**Affiliations:** 1 Department of Pulmonology and Allergy, Soonchunhyang University Cheonan Hospital, Cheonan 31151, Korea; euphoria97@naver.com; 2 Department of Psychiatry, Soonchunhyang University Cheonan Hospital, Cheonan 31151, Korea

**Keywords:** electroencephalography, event-related potential, precision medicine, prediction modeling, biomarker

## Abstract

Precision medicine has been considered a promising approach to diagnosis, treatment, and various interventions, considering the individual clinical and biological characteristics. Recent advances in biomarker development hold promise for guiding a new era of precision medicine style trials for psychiatric illnesses, including psychosis. Electroencephalography (EEG) can directly measure the full spatiotemporal dynamics of neural activation associated with a wide variety of cognitive processes. This manuscript reviews three aspects: prediction of diagnosis, prognostic aspects of disease progression and outcome, and prediction of treatment response that might be helpful in understanding the current status of electrophysiological biomarkers in precision medicine for patients with psychosis. Although previous EEG analysis could not be a powerful method for the diagnosis of psychiatric illness, recent methodological advances have shown the possibility of classifying and detecting mental illness. Some event-related potentials, such as mismatch negativity, have been associated with neurocognition, functioning, and illness progression in schizophrenia. Resting state studies, sophisticated ERP measures, and machine-learning approaches could make technical progress and provide important knowledge regarding neurophysiology, disease progression, and treatment response in patients with schizophrenia. Identifying potential biomarkers for the diagnosis and treatment response in schizophrenia is the first step towards precision medicine.

## 1. Introduction

Precision medicine is increasingly recognized as a promising approach to improve disease prevention, diagnosis, and treatment, considering the individual clinical and biological characteristics shared by specific subgroups of patients [1]. It considers the variability in patient and disease characteristics, genes, environment, and lifestyle of each person [2].

Individualized prediction models have been shown in psychiatry, and a new department of precision psychiatry has come out [2]. Despite the growing interest and clinicians’ efforts to find alternatives, diagnosis and treatment have relied heavily on clinical interviews rather than direct, reliable assessments of brain function, and we could not have improved clinically reasonable points for various psychiatric disorders over the past years [3]. Recent advances in biomarker development hold promise for guiding a new era of precision medicine style trials for psychiatric disorders [3].

The launch of the Research Domain Criteria (RDoC) project of the National Institute of Mental Health (NIMH) to address the need for a new approach to classifying mental disorders has given strength to the movements of precision medicine. The RDoC project was an approach that would begin with, but not be limited to, symptoms [4].

Compared to classical precision medicine, modern advancements in knowledge in the field of individualized prediction modeling have allowed the consolidation of an evidence-based science of precision medicine [5]. Prediction modeling can be used to predict the probability of diagnosis, disease progression (outcome), and treatment response [2]. In particular, individualized prediction models have been extensively investigated in psychiatry related to psychosis [2]. Moreover, prediction models incorporate a clinical staging model for psychosis [6], and research on prognostic prediction models related to the emergence of a clinical high-risk state for psychosis [7] has been proposed [8].

Electroencephalography (EEG) allows noninvasive assessment of cognitive processes in real time [9]. The great time resolution of EEG can evaluate fast neural events and allows for recognizing the associated brain area through source analysis [9]. EEG can follow the neural activation associated with multiple cognitive tasks [10]. Event-related potentials (ERPs) enable a temporally detailed exploration of the cognitive process; early components of ERP reflect sensory or perceptual processing, and later components reflect higher cognitive processes [11]. Specifically, ERPs could be an effective tool for verifying brain synaptic functions [12,13,14]. Because ERP allows us to know the temporal patterns of neuronal activity, it can be useful in quantifying the sequence of various cognitive processes [13].

In addition, from a practical perspective, it is important to have simple paradigms, especially for patients with psychosis. The ERP stimuli are quite simple, such as a two- or three-tone auditory oddball paradigm [15]. EEG could be useful for evaluating patients with psychotic symptoms because it is less influenced by movement artifacts than other brain investigating tools. Moreover, EEG is an inexpensive and noninvasive tool for the evaluation of psychiatric pathophysiology; thus, it is available for both research and clinical assessment for patients suffering from psychological distress.

Owing to these methodological advantages, several EEG biomarkers have been extensively evaluated in psychosis. Among them, mismatch negativity (MMN) is a well-known EEG marker for schizophrenia associated with neurocognitive impairment in psychosis. A reduction in auditory MMN amplitude was reported over two decades ago in patients with schizophrenia [16]. It has been replicated numerous times as a mature neurophysiological biomarker based on criteria such as scalability, low cost, and suitability for use in multicenter studies [3].

This review examines three aspects that might be helpful in understanding the current status and possibility of using electrophysiological biomarkers in precision medicine for patients with psychosis: (1) the prediction of diagnosis, (2) the prognostic aspects of disease progression and outcomes, and (3) the prediction of treatment response. In addition, the review also describes clinical and research implication of electrophysiological approaches as useful biomarkers.

## 2. EEG Biomarkers for Predicting Diagnosis of Psychosis

As mentioned, MMN is a frequently studied ERP in schizophrenia. MMN is a measure of automatic neurophysiological brain processes for detecting unexpected sensory stimuli [17]. It is also an ERP elicited when a sequence of unattended repetitive sounds is interrupted by a deviant stimulus [18]. The critical pathology of MMN attenuation might have originated from the abnormality of the N-methyl-D-aspartate (NMDA) receptor system [19]. Both patients with schizophrenia [20] and bipolar disorder [17] have MMN reduction and it may be well explained by NMDA-receptor-mediated glutamatergic dysfunction. Kim et al. showed that both patients with schizophrenia and bipolar disorder exhibited significantly reduced MMN amplitudes compared with the healthy population [17]. Specifically, patients with bipolar disorder had intermediate MMN amplitudes among the groups [17]. The cortical thickness of the right superior temporal gyrus were significantly negatively correlated in patients with schizophrenia [17], suggesting that ERP could offer critical information about the associated brain areas of ERP components in schizophrenia. However, considering that both schizophrenia and bipolar disorder attenuate MMN amplitude, it is difficult to draw conclusions that MMN could be a biomarker to predict psychosis diagnosis.

Although EEG analysis has appeared as a powerful method for brain state interpretation and diagnosis, but not for the diagnosis of psychiatric illness, it might be accounted by its low depth sensitivity and spatial resolution [21]. However, recent methodological advances have shown the possibility of classifying and detecting mental illnesses. One recent study that investigated abnormal neural oscillations, which have been shown to precede the onset of frank psychosis, could be used for individualized prediction of psychosis in clinical high-risk patients [22]. The study also assessed the individualized prediction of psychosis by detecting specific patterns of beta and gamma oscillations using machine-learning algorithms [22]. Transition to psychosis could be predicted from current–source density in the trained, and tested on 53 neuroleptic-naïve patients with a clinical high-risk for psychosis [22]. The study revealed that the left superior temporal gyrus, left inferior parietal lobule, and precuneus most strongly contributed to the prediction of psychosis [22]. Therefore, these results suggest that current–source density measurements extracted from clinical resting-state EEG can help improve the prediction of psychosis [22].

Another recent study with a novel approach for schizophrenia detection introduced the time-frequency transformation followed by feature optimization, which showed great success in classification accuracy with no false positives [21]. The study revealed a high accuracy for data classification, with a prediction accuracy of the five distinctive electrodes between 91.5% and 93.9% with the best frontal electrode such as F2 [21].

## 3. EEG Biomarkers for Disease Progression and Outcome in Psychosis

P50 suppression impairment has been regarded as intermediaries between the molecular mechanisms and clinical characteristics of schizophrenia [23]. Sensory processing deficits are the basis of complex cognitive dysfunction and are influenced by difficulties of higher cognitive function [23]. In addition, P50 has been considered a possible stable trait marker in psychosis unaffected by treatment with medication [23]. Previous studies have illustrated the effective role of P50 suppression in the early detection and prediction of schizophrenia [24]. Based on this knowledge, Luo and colleagues examined the effects of sensory gating on the performance of 136 participants in a P50 sensory gating task, including patients with first-episode schizophrenia, ultrahigh-risk individuals, high-risk individuals, and a healthy general population using EEG [24]. Compared with the healthy population, the other groups showed significant P50 suppression impairment [24]. Furthermore, EEG source localization analyses showed successively stronger activation in the prefrontal and anterior temporal regions in the schizophrenia and at-risk groups than in the healthy population [24]. More interestingly, brain connectivity in the gamma band of P50 components was increasingly enhanced in accordance with the level of psychosis risk [24]. These results suggest that EEG source imaging techniques and brain network dynamics can clearly distinguish the different stages of psychosis.

Recently, longitudinal studies to confirm the possibility of biomarkers related to psychosis transition have been conducted. One recent study compared participants with clinical high-risk for psychosis who converted to psychotic disorder versus those who did not convert to psychosis [25]. Individuals who converted to psychosis had attenuated MMN compared to those with nonconversion [25]. The other study also revealed that reduced MMN could predict the onset of psychosis in clinical high-risk individuals [26]. It suggests that MMN could be a useful biomarker to forecast the development of psychosis and estimate the time lag to psychosis onset [27].

MMN is known to be associated with social cognition and functional outcomes [28]. Previous studies have reported that greater MMN activity correlates with better productivity in the workplace and independent living, and with better social perception in patients with schizophrenia [28]. In addition, MMN deficits represent a core neurophysiological dysfunction that is linked to global impairments in everyday functioning in schizophrenia patients [29]. Light and Braff’s study showed that greater levels of MMN impairment were associated with lower Global Assessment of Functioning Scale ratings in patients with schizophrenia [29]. A regional analysis of MMN revealed that the largest correlations of MMN to everyday functioning were present at frontocentral electrode sites [29].

The MMN component also identified a possible predictive marker for illness outcomes. A recent study with 48 patients at clinical high risk for psychosis participated in the MMN assessment showed that nonremitters showed reduced MMN amplitudes at baseline compared to remitters [25]. In addition, MMN amplitude at the frontal site was the only meaningful predictor of remission [25]. This suggests that MMN is a putative predictor of prognosis, regardless of the transition to psychotic disorder in subjects at clinical high risk [25]. Furthermore, a clinical high risk for psychosis and a population already diagnosed with schizophrenia also showed MMN attenuation associated with remission. Another with 40 participants with schizophrenia assessed with MMN showed that nonremitters showed reduced MMN amplitudes in frontal sites compared to remitters [26]. Moreover, MMN amplitude was significantly correlated with measures of symptom change and functional outcome measurements in patients with schizophrenia [26].

Regarding the functionality of schizophrenia as an important prognostic factor of psychosis, 25 patients with schizophrenia had significantly reduced MMN, and greater levels of MMN impairment were associated with lower Global Assessment of Functioning Scale ratings [27]. In this study, the largest correlations of MMN with everyday functioning were present at frontocentral recording sites [27]. These results suggest that MMN deficits represent a core neurophysiological dysfunction linked to global impairments in everyday functioning in patients with schizophrenia [27].

Interestingly, MMN deficits may index both ongoing disease processes associated with illness progression, as well as with premorbid neurocognitive impairment [15,28]. Additionally, impaired MMN in patients with schizophrenia and its association with impaired functional status have been consistently reported [29,30]. Regarding the association of MMN with neurocognition, functioning, and illness progression in schizophrenia, it could be speculated that MMN affects remission and recovery of schizophrenia [26].

## 4. EEG Biomarkers for Treatment Response in Psychosis

Recently, machine learning has allowed researchers to obtain knowledge from EEG, which can be useful in predicting treatment response or determining the site of action of antipsychotics [30].

A resting EEG study with treatment-resistant schizophrenia showed quantitative electroencephalography (QEEG) activity during auditory hallucinations [31]. QEEG is a methodology that processes the recorded EEG activity from a multielectrode recording using a computer [31]. This multichannel EEG data are processed with mathematical algorithms, such as the Fourier transformation. These processed EEG data are converted into brain maps [31]. The study also evaluated increased phase coupling of theta and gamma frequencies in the left frontotemporal area before experiencing hallucination [31]. A study with QEEG-based neurofeedback with elevated theta activity as a target revealed 82% improvement in psychotic symptom scores with one-hour neurofeedback sessions in 48 patients with schizophrenia [32].

Khodayari et al. developed machine-learning algorithms capable of predicting the response to clozapine with 85% accuracy [33]. Machine-learning techniques are finding increasing application in psychiatry, particularly when multidimensional, noisy, highly complex data are analyzed together [33]. In this study, the predictive features selected by algorithms were coherences between the left frontal and parietal leads and left temporal leads in the 6–13 Hz range [33]. In addition, using machine-learning algorithms with brain localization methods, Ravan et al. further assessed the relationship between EEG and clozapine treatment response to examine P300 responses with the auditory oddball paradigm in 47 patients with schizophrenia [34]. They also identified several source generators of P300 that changed after clozapine treatment [34]. Specifically, the central and right temporal regions were differentiated schizophrenia patients from a healthy population with 84% accuracy [34]. These results suggest that machine-learning analysis of EEG may be a useful method for evaluating treatment response in psychotic disorders.

P50 suppression has been associated with cognitive impairment in schizophrenia [35]. In one study, tropisetron, a novel α7 nicotinic acetylcholine receptor agonist, normalized P50 suppression deficits in patients with schizophrenia [36]. At the same time, the patients showed improved cognition with medication, implying that deficits in P50 suppression in patients with schizophrenia might be a biomarker for future trials of cholinergic agents for the treatment of cognitive deficits [36].

Regarding the cognitive impairment observed in patients with schizophrenia, many studies have attempted to use procognitive drugs to attenuate cognitive decline [37]. For example, there has been great interest in the N-methyl-D-aspartate receptor (NMDA) receptor antagonist, memantine, which has been approved for Alzheimer’s disease [38]. Interestingly, auditory MMN is thought to be an index of NMDA function [39]. Light et al. found that memantine enhanced MMN in patients with schizophrenia, and MMN was associated with less cognitive decline and greater psychosocial functioning [3,40]. This suggests that MMN might be a biomarker of treatment engagement in cognitive interventions for psychotic disorders.

Besides pharmacologic treatment, MMN may be also sensitive to cognitive therapy such as targeted cognitive training [27]. It is computerized cognitive exercises and it targets the accuracy of auditory sensory information processing and working memory [41]. Several studies to be conducted for evaluating the possibility of MMN to predict response of targeted cognitive training. For example, the study of a three-week intensive auditory-frequency-discrimination training produced significant increases in MMN [27,42]. Another study showed that MMN predicts improvement in auditory-dependent cognitive tasks in participants [43]. Most studies revealed that higher baseline MMN predicted better outcome [27].

## 5. Clinical Implication of EEG Biomarkers in Psychosis

The development of biomarkers for psychosis should be used for proper treatment and improvement of prognosis in patients. Specifically, for the EEG biomarkers to be used in actual clinical settings, various obstacles must be resolved. First is the matter of false positives and proper validation of electrophysiological components [27]. Moreover, the methods need to be simplified to allow administration by nonpsychiatrists in actual clinical settings [27]. To solve these problems, Light and his colleagues conducted the study that tested the feasibility of adding MMN and P300 to the Consortium on the Genetics of Schizophrenia (COGS) study [44]. In the study, there were significant correlations among demographically adjusted MMN and P3a and several clinical, cognitive, and functional characteristics of the patients with schizophrenia. It reveals that MMN and P300 biomarkers can be feasibly used in multisite clinical studies. The study suggests that ERP biomarkers such as MMN and P300 and should be carefully considered in future biomarker-informed clinical studies. Light’s study took the first step to show the possible feasibility and scalability of EEG by not detecting site differences. Such ready scalability is necessary to solve the problem of site difference.

## 6. Research Implication of EEG Biomarkers in Psychosis

Debilitating mental states comprise psychotic disorder, which affect various domains of daily functioning [45]. Early detection and timely treatment are crucial to improve clinical and functional outcome in psychotic disorder [46]. Based on this concept, extensive research has been conducted to identify biomarkers in patients with psychosis. Specifically, regarding ultrahigh risk (UHR) patients who transition to psychosis, many researchers tried to detect transition prediction to psychosis [47]. For example, resting-state EEG microstates [47] and MMN [27] have been candidate biomarkers to detect transition from UHR to psychosis. Although these studies to find EEG markers for the diagnosis of psychosis have been conducted, evidence of basic experimental models to predict psychotic condition using EEG should be developed for the gain of clinical validity. MMN has been explored not only in clinical samples but also in basic research [48]. Many studies have reported MMN in rat and mouse models that are already referred to in a comprehensive review [49]. Moreover, Javitt and colleagues have reported that NMDA receptor antagonists reduced MMN amplitude in the awake, nonhuman primate [50]. However, it is unclear how the injection dosage used in the study associated with the subanesthetic injection that induces psychosis in humans [48]. Therefore, further studies are needed to clarify the cellular mechanism of MMN for diagnosis or treatment based on the molecular hypothesis of schizophrenia.

Apart from the fact that MMN and other EEG marker research could contribute to diagnostic and therapeutic strategies in the future, finding the biomarkers for psychosis is very complicated. For example, schizophrenia is a neurodevelopmental disorder that is affected by genetic events; additionally, it is affected by environmental influences after birth [27]. Therefore, Light and colleagues proposed further development of biomarkers for predicting treatment response [27]. Regarding the precision medicine for psychosis, their proposal might be consistent with the therapeutic goals of personalized medicine.

## 7. Conclusions

Precision medicine is increasingly recognized as a promising approach to improve disease prevention, diagnosis, and treatment, and individualized prediction models have been developed in the new field of precision psychiatry. In particular, these individualized prediction models have been extensively investigated for psychotic disorders. Among biological methodologies, EEG provides a direct measure of neural activity and high temporal resolution. In addition, it has cost advantages and good accessibility. Investigating ERPs in particular is a noninvasive method for assessing spatiotemporal activation in the brain during sensory, cognitive, and affective processing in real time. Previous studies that we reviewed reveal the possibility of EEG as a research method for precision medicine in patients with schizophrenia. Resting state study, sophisticated ERP measures, and machine-learning approaches could make technical progress and provide important knowledge regarding neurophysiology in patients with schizophrenia. This study is limited to a narrative review; further systemic review or meta-analysis related to electrophysiological biomarkers for psychosis is needed. Moreover, other advanced techniques such as imaging studies should be reviewed in future studies. Identifying potential biomarkers for the diagnosis and treatment response in schizophrenia is the first step toward precision medicine. The discovery of biomarkers is an essential element for the development of an objective means to confirm the diagnosis and determine optimal treatment for psychotic disorders.
